# Extensive myelitis with eosinophilic meningitis after Chimeric antigen receptor T cells therapy

**DOI:** 10.1002/jha2.381

**Published:** 2022-02-01

**Authors:** Baptiste Le Calvez, Marion Eveillard, Paul Decamps, Jesus Aguilar, Amélie Seguin, Emmanuel Canet, Audrey Grain, Cyrille Touzeau, Benoît Tessoulin, Thomas Gastinne

**Affiliations:** ^1^ Department of Hematology Nantes University hospital Nantes France; ^2^ Nantes Université, INSERM, CNRS, Université d'Angers, CRCI2NA Nantes France; ^3^ Site de Recherche Intégrée sur le Cancer, ILIAD INCA‐DGOS‐Inserm U12558 Nantes France; ^4^ Hematology Biology Nantes University Hospital Nantes France; ^5^ Intensive Care Unit Nantes University Hospital Nantes France; ^6^ Medical Imaging Unit Nantes University Hospital Nantes France; ^7^ Pediatric Oncology Nantes University Hospital Nantes France

**Keywords:** Chimeric antigen receptor T cells, eosinophilic meningitis, eosinophilic pleocytosis, mantle cell lymphoma, myelitis

## Abstract

Immune effector cell‐associated neurotoxicity syndrome (ICANS) is a frequent adverse event after Chimeric antigen receptor T cells (CAR‐T cells). A patient treated with anti‐CD19 CAR‐T cells for a refractory mantle cell lymphoma presented at Day 8 post‐infusion with extensive myelitis. Unusual eosinophilia was disclosed in the patient's cerebrospinal fluid. After treatment with methylprednisolone and siltuximab, a decrease in clinical symptoms and magnetic resonance imaging lesions were obtained. This unprecedented presentation of eosinophilic meningitis after CAR‐T cells therapy highlights the need for a better understanding of the physiopathology of ICANS, especially to identify potentially targetable pathways.

## 1 INTRODUCTION

Chimeric antigen receptor T cells (CAR‐T cells) have revolutionized the management of several hematologic malignancies in recent years [1, 2, 3]. However, this immunotherapy is associated with significant side effects. Neurological toxicity, formerly called CAR‐related encephalopathy syndrome and now termed immune effector cell‐associated neurotoxicity syndrome (ICANS), is the second most common adverse event after cytokine release syndrome (CRS) [4]. Encephalopathy is by far the most frequent expression of ICANS. Its evolution is frequently stereotyped with the successive appearance of tremor, phasic and graphic disorders, followed by vigilance disorders [4]. Various symptoms have been reported, and, therefore, any neurological sign occurring after the CAR‐T cells infusion should be considered as ICANS until proven otherwise. An unusual case of ICANS with eosinophilic involvement is reported here.

## 2 CLINICAL PRESENTATION

A 52‐year‐old Caucasian male with mantle cell lymphoma resisted four lines of systemic therapy including chemotherapy and autologous hematopoietic stem cell transplantation. Prior to anti‐CD19 CAR T‐cell therapy, the patient received three courses of obinutuzumab plus bendamustine which did not result in a significant metabolic or morphological response. The pre‐CAR‐T assessment showed a high tumor burden with the main mass measured at 124 × 54 mm. Pre‐therapy brain magnetic resonance imaging (MRI) showed no significant abnormality except for a 14 mm right anterior temporal arachnoid cyst without mass effect. There was no history of neuromeningeal involvement. There was no biological inflammatory syndrome before CAR T‐cells infusion. The patient received CAR‐T cells (brexucabtagene autoleucel) after conventional lymphodepletion with fludarabine and cyclophosphamide.

**FIGURE 1:**
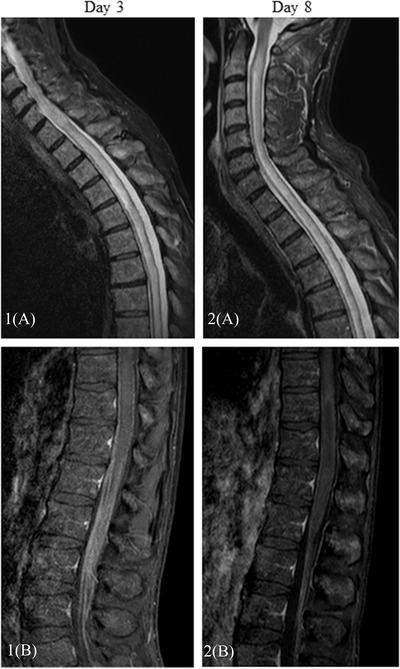
(1A) D3 magnetic resonance imaging (MRI)/sagittal short tau inversion recovery (STIR) images: extensive T2 hypersignal of the medulla, tumor‐like appearance. (1B) D3 MRI/sagittal T1 fat‐saturated post‐contrast images: poorly delineated thoracic medulla contrast patches. Clear contrast of the roots of the cauda equina. (2A) D8 MRI/sagittal STIR images: decrease of the tumescent aspect of the medulla. Stability of the T2 hypersignal extent of the medulla. (2B) D8 MRI/sagittal T1 fat saturated post‐contrast images: significant decrease in medullary contrast, especially in the roots of the cauda equina

**FIGURE 2:**
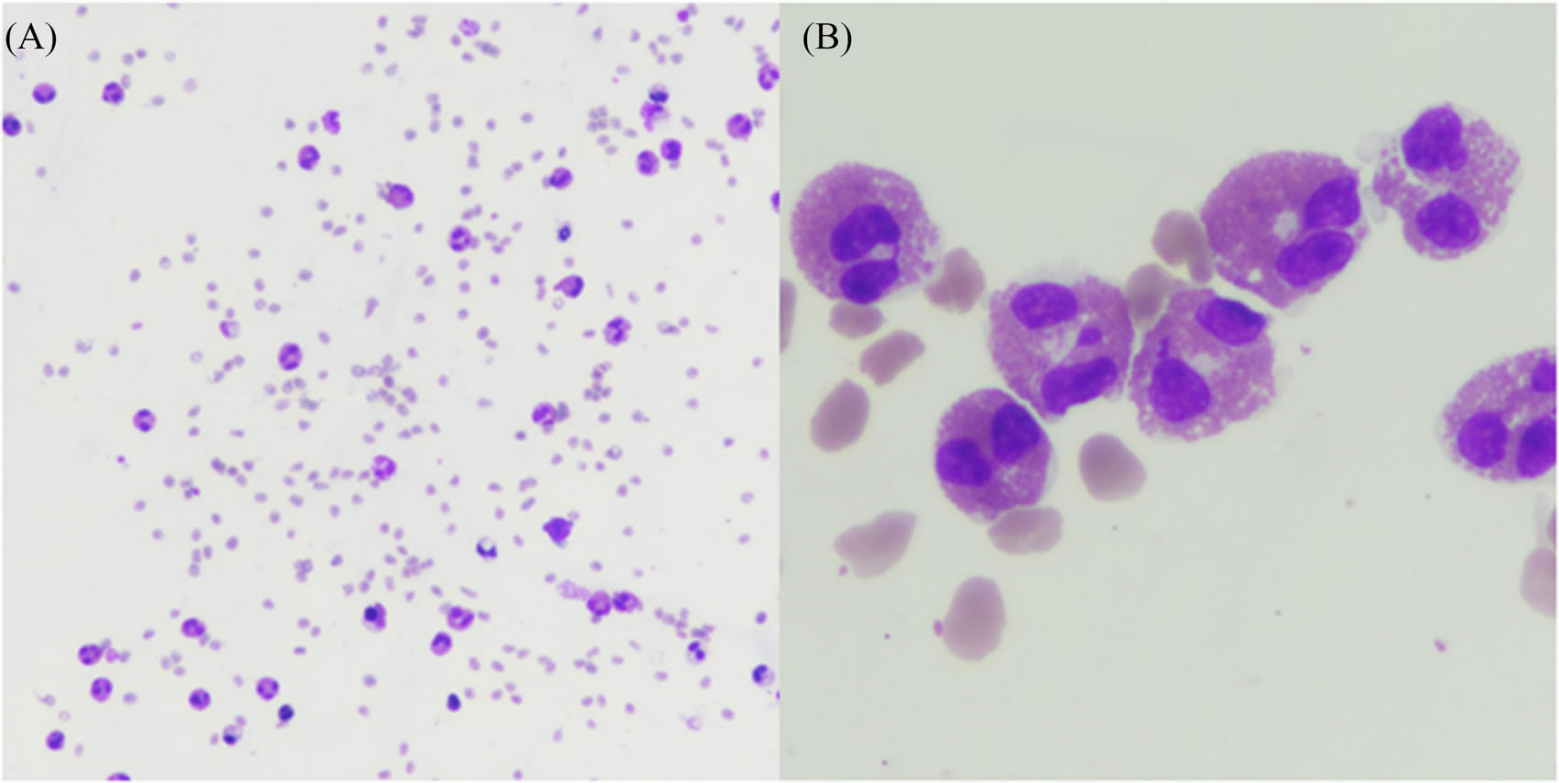
Cerebrospinal fluid (CSF) May Grunwald Giemsa cytospin stain, magnification 10x (A) and 50x (B)

From Day 1 after CAR T‐cells infusion, the patient experienced grade 1 CRS with fever alone. On Day 2, a probabilistic antibiotic treatment with cefepime was introduced, as well as a single dose of tocilizumab due to the persistence of fever associated with tachycardia, together with anti‐epileptic prophylaxis with levetiracetam. On Day 3, the patient presented a complete and symmetrical motor deficit of the lower limbs associated with areflexia. Progressively, anesthesia of the lower limbs appeared, associated with saddle anesthesia and sphincter disorders. The patient did not show any sign of encephalopathy, the ICE score was evaluated at 10/10. The same day, a cerebral and medullary MRI was performed. It disclosed extensive myelitis associated with infiltration of cauda equina roots (Figure 1). Brain MRI showed the appearance of a lesion in front of the right lenticular nucleus, with gadolinium enhancement. Cerebrospinal fluid (CSF) analysis disclosed 252 elements per mm^3^ with 92% of eosinophilic polymorphonuclears (Figure 2). There was also hypoglycorrhachia at 1.4 mmol/L. Microbiological investigations did not reveal any evidence of infectious meningitis (bacteriological and mycological cultures, CSF cryptococcal antigen assessment, CSF 1,3‐β‐d‐glucan, CSF galactomannan antigen, molecular test for herpes simplex virus, varicella‐zoster virus, enterovirus, parechovirus, John Cunningham virus, cytomegalovirus, human herpesvirus 6, and toxoplasma). Toxocariasis serology was negative, and the patient had never traveled abroad. There was no peripheral blood eosinophilia. No other organ damage, particularly cardiac, was identified. Antibiotics were continued and dexamethasone 20 mg every 6 h was started on Day 3. Given the progressive extension of the motor deficit, affecting the upper limbs from Day 4, treatment with methylprednisolone 1 g per day was carried out from Day 4 to Day 6, associated with a single dose of siltuximab 11 mg/kg at Day 6. The progression of neurological symptoms stopped as of Day 7. A new analysis of the CSF at that time showed a decrease of the cell infiltration down to six elements per mm^3^ still composed of 77% of eosinophilic polymorphonuclears, associated with a normalization of glycorrhachia. The MRI performed on Day 8 showed a clear decrease of contrast enhancement on the whole spinal cord with the persistence of an extensive Short tau inversion recovery hypersignal. A regression of the contrast enhancement of cauda equina roots was described. In addition, a decrease in Fluid‐attenuated inversion recovery hypersignal of the lenticular nucleus was noted. From Day 7 to Day 11, the neurological symptoms remained stable without improvement or deterioration. Unfortunately, the patient died on Day 11 from an *E. coli* bloodstream infection.

## 3 DISCUSSION

Eosinophilic meningitis can have several etiologies [5]. Most of them are infectious, related to parasitosis or fungal infection, and exceptionally to bacteria or viruses [6]. It can also be related to hematological malignancies, such as Hodgkin's disease, or allergic or autoimmune disorders. Neurological toxicities have been described with fludarabine, but are rare, delayed, and progressive [7]. To our knowledge, no myelitis with eosinophilic meningitis has been described with fludarabine.

The mechanism of ICANS remains largely ill‐understood but is partly explained by disruption of the blood‐brain barrier, brain edema, and endothelial activation via cytokine release from CAR‐T cells [8]. Several studies have highlighted the presence of CAR‐T cells and inflammatory cytokines in the CSF of patients with ICANS [9, 10]. However, there appears to be no correlation between white blood cells count and the amount of CAR‐T in CSF nor with the presence or severity of ICANS [11]. In most cases, the CSF is discreetly inflammatory with hyperproteinorachia, usually without major pleocytosis [10, 11, 12].

Several cases of myelitis after CAR‐T cells therapy have been reported, but none of them was associated with eosinophilic pleocytosis [13, 14]. Although no cases have been reported yet, Santomasso et al. showed that plasma Interleukin 5 levels were significantly associated with the severity of neurotoxicity in a series of 53 adult patients with acute lymphoblastic leukemia treated by CAR‐T cells [11]. Peripheral hypereosinophilia can be responsible for various tissue lesions, owing to the extremely basic content of eosinophilic granules, and this case of myelitis raises the question of the possibility of direct eosinophil toxicity on the spinal cord [15].

This highlights the need for a better understanding of the physiopathology of ICANS, especially to identify potentially targetable pathways, in these peculiar situations.

## CONFLICT OF INTEREST

The authors declare that they have no conflict of interest.

## AUTHOR CONTRIBUTIONS

Baptiste Le Calvez wrote the manuscript. Marion Eveillard performed biological analyses. Baptiste Le Calvez, Cyrille Touzeau, and Paul Decamps treated the patient. Jesus Aguilar provided imaging data. All authors critically reviewed the manuscript.
